# Effects of *Bifidobacterium* with the Ability of 2′-Fucosyllactose Utilization on Intestinal Microecology of Mice

**DOI:** 10.3390/nu14245392

**Published:** 2022-12-19

**Authors:** Bingyong Mao, Zhujun He, Yang Chen, Catherine Stanton, Reynolds Paul Ross, Jianxin Zhao, Wei Chen, Bo Yang

**Affiliations:** 1 State Key Laboratory of Food Science and Technology, Jiangnan University, Wuxi 214122, China; 2 School of Food Science and Technology, Jiangnan University, Wuxi 214122, China; 3 International Joint Research Center for Probiotics & Gut Health, Jiangnan University, Wuxi 214122, China; 4 APC Microbiome Ireland, University College Cork, T12 R229 Cork, Ireland; 5 Teagasc Food Research Centre, Moorepark, Co., P61 C996 Cork, Ireland; 6 National Engineering Research Center for Functional Food, Jiangnan University, Wuxi 214122, China

**Keywords:** *Bifidobacterium*, 2′-fucosyllactose, intestinal microecology, mixed strains

## Abstract

In breast milk, 2′-Fucosyllactose (2′FL) is the most abundant breast milk oligosaccharide and can selectively promote the proliferation of bifidobacteria. This study aimed to explore the effect of ifidobacterial with different utilization capacities of 2′FL on the intestinal microecology of mice. Furthermore, the effects of ifidobacterial with different 2′FL utilization capabilities on mice gut microbiota under the competitive pressure of 2′FL as a carbon source were explored. Compared with the control group, 2′FL, *Bifidobacterium* (*B.*) *bifidum* M130R01M51 + 2′FL, *B*. *longum* subsp. *Longum* CCFM752, and CCFM752 + 2′FL treatments significantly decreased the food intake. Moreover, the water intake, body weight, and fecal water content in all groups showed no significant difference compared with the control group. The combination of *B. longum* subsp. *longum* CCFM752 and 2′FL can significantly increase the levels of pro-inflammatory and anti-inflammatory factors. *B. bifidum* M130R01M51 and mixed strains combined with 2′FL significantly increased the contents of acetic acid and isobutyric acid. The results showed that *B. bifidum* M130R01M51, *B. breve* FHuNCS6M1, *B. longum* subsp. *longum* CCFM752, and *B. longum* subsp. *infantis* SDZC2M4 combined with 2′FL significantly increased the species richness of the gut microbiota. Moreover, *B. longum* subsp. *longum* CCFM752 and *B. longum* subsp. *infantis* SDZC2M4 significantly increased the abundance of *Faecalibaculum* and *Bifidobacterium*, respectively. In conclusion, exploring the impact on intestinal microecology can provide theoretical guidance for the development of personalized prebiotics for different bifidobacteria, which has the potential to improve the ecological imbalance of infant gut microbiota.

## 1. Introduction

Gut microbiota is involved in physiological functions such as metabolism, nutrition, and immunity and plays a very important role in human development [1]. In general, bifidobacteria predominate in the microbiota of the infant gut and are more prevalent in the gut microbiota of breast-fed infants than in formula-fed infants [2,3]. Moreover, breast-fed infants have shown decreased risks for pathogenic infection and allergies than formula-fed infants [4,5].

Human milk oligosaccharides (HMOs) are a class of complex mixed oligosaccharides that play key roles in infant growth and development [6,7,8] and are mainly composed of glucose, galactose, *N*-acetylglucosamine, fucose, and sialic acid [9]. The most common HMOs are 2′-fucosyllactose (2′FL), 3′-fucosyllactose (3′FL), 3′-sialyllactose (3′SL), 6′-sialyllactose (6′SL), lactose-n-tetraose (LNT), and lactose-n-neotetraose (LNnT). In the human body, HMOs are not digested by digestive enzymes in the small intestine, and almost all of them are utilized by microorganisms in the large intestine [10]. Moreover, 2′FL is the most abundant fucosylated HMO, which selectively promotes the colonization of *Bifidobacterium* [10]. It is composed of fucose and galactose linked by α-1,2 glycosidic bonds but is only found in secreted breast milk [11]. For breast-fed newborns, bifidobacteria could metabolize 2′FL to colonize in the intestinal tract [12]. Furthermore, short-chain fatty acids (SCFAs), such as acetate produced by *Bifidobacterium* from oligosaccharides, inhibit inflammation and allergic reactions by inducing the differentiation of colon regulatory T cells (Treg) [13,14] and enhancing the number and function of lung Treg cells [15]. It can be concluded that bifidobacteria in the intestinal tract of breast-fed infants could degrade and utilize 2′FL, thus maintaining intestinal immune homeostasis and promoting intestinal health.

*B. bifidum* could hydrolyze 2′FL by exocrine fucosidase. Other bifidobacteria mostly rely on ABC transporters to transport 2′FL into the cell, and then use various glycoside hydrolases to complete the utilization of 2′FL [16], while *Bifidobacterium* can synthesize fucosidase (GH95 and GH29), β-galactosidase, and other glycosidolytic enzymes [17,18]. β-galactosidase is widely found in *Bifidobacterium*; therefore, the utilization of 2′FL by bifidobacteria mainly depends on the fucosidase gene [18]. Hence, whether *Bifidobacterium* strains contain an active fucosidase gene and ABC transporter are important to determine their abilities to use 2′FL.

To date, many studies have evaluated the metabolizing ability of certain *Bifidobacterium* species to 2′FL and have found that *B. longum* subsp. *infantis*, *B. bifidum*, and *B. breve*, as well as *B. longum* subsp. *longum*, can grow well with 2′FL as the sole carbon source [6,16,19,20,21]. These are also the most common bifidobacteria found in the intestines of breast-fed infants. In addition, other studies have demonstrated that *B. kashiwanohense* could utilize 2′FL but could not utilize L-fucose, which was released by the intracellular hydrolysis of 2′FL [22]. *B. pseudocatenulatum* LH13, an infant-derived strain, contains a well-known fucosylated HMOs utilization gene cluster, which can encode the ABC transporter and key GH95 gene [23]. Recently, BSM11-5 of *B. longum* subsp. *suis* has also been reported to utilize 2′FL to a certain extent despite its low abundance in the infant gut [22].

It has been reported that probiotics combined with indigestible oligosaccharides can better balance the gut microbiota, while multi-strain preparations may show higher functionality than single-strain cultures [24,25]. The current research on the utilization of 2′FL by *Bifidobacterium* is mainly focused on the evaluation of key genes, utilization ability, and utilization mechanism in a single strain [6,10,16,26,27]. However, the research on *Bifidobacterium* combined with 2′FL on the intestinal microecology of a healthy host is relatively scarce. Therefore, this study aimed to investigate the effects of bifidobacteria with different utilization abilities of 2′FL on the intestinal environment, which will provide support for the development of 2′FL as new-generation prebiotics.

## 2. Materials and Methods

### 2.1. Bifidobacterium Strains and Culture Conditions

Five *Bifidobacterium* strains with different utilization abilities of 2′FL were used in this study, including *B. bifidum* M130R01M51, *B. breve* FHuNCS6M1, *B. longum* subsp. *longum* CCFM752, *B. longum* subsp. *infantis* SDZC2M4, and *B. dentium* FJSWXJ29M2. Their abilities to utilize 2′FL were confirmed in our previous work [28]. All the strains were deposited in the Culture Collection of Food Microorganisms (CCFM) of Jiangnan University (Wuxi, China).

All five *Bifidobacterium* strains were anaerobically cultured at 37 °C for 24 h and centrifuged at 8000× *g* for 20 min; then, the supernatant was removed, and the cell pellets were washed with 0.9% normal saline (added with 0.05% l-cysteine). Then, the washed bacterial sludge was added to the prepared sucrose protective agent (sterile 30% sucrose solution) at a ratio of 1:2. The samples were packed into 1.5 mL Eppendorf tubes and stored at −80 °C. In the experiment, the bacterial solution was diluted to 5 × 10^9^ CFU/mL with 0.9% normal saline.

### 2.2. Animal Experimental Design

One hundred and twelve C57BL/6J mice (male, 3 weeks old) were obtained from the Institute of Model Zoology, Nanjing University (Nanjing, Jiangsu, China) and raised in the experimental animal center of Jiangnan University. They were randomly divided into 14 groups with 8 mice in each group (Table 1), and 4 mice in the same group were kept in one cage. The environment temperature (20–26 °C) and humidity (40%–70%) were strictly controlled. Each mouse was orally gavaged with different composition of 200 μL from day 8 to 28. All mice had ad libitum access to water and food. The food intake, water intake, and body weight of the mice were recorded every week. The experimental design is shown in Table 1. The procedures of animal experiment was approved by the Ethics Committee of Experimental Animals at Jiangnan University (qualified number: JN.No20200710c1041015[162]]).

### 2.3. Histopathological Examination

A histopathological examination was performed on intestinal tissue. After dissection, intestines were fixed in 4% formalin, embedded in paraffin, and stained with hematoxylin and eosin (HE). All sections were observed under a digital slide scanner microscope (Pannoramic MIDI II, 3DHISTECH Ltd, Hungary).

### 2.4. Cytokines in Colon Tissue

The colonic concentrations of IL-6, IL-1β, TNF-α, IL-10, and IL-4 were measured by commercial ELISA kits following the manufacturer’s instructions (R&D Systems Co., Ltd., Minneapolis, MN, USA).

### 2.5. Short-Chain Fatty Acids Analysis

The extraction and pretreatment methods of fatty acids in colon tissue were performed according to previous methods [29,30]. Then, short-chain fatty acids were determined by GC-MS, and the detailed parameters of GC-MS were performed as described previously [31,32].

### 2.6. Intestinal Microbiota Analysis

Genomic DNA was extracted from frozen feces using a Fast DNA Spin Kit for Feces (MP Biomedicals, LLC, Irvine, CA, USA) following the manufacturer’s instructions. The V3–V4 region of 16S rRNA was PCR-amplified using primers as in the previous description [33]. To further distinguish the species of *Bifidobacterium*, the *groEL* gene was amplified according to the method described by Mao et al. [34]. The PCR products were purified by the DNA gel/PCR purification miniprep Kit (Biomiga, San Diego, CA, USA). After sequencing, QIIME 2 was used to analyze the sequence data. Linear Discriminant Analysis Effect Size (LEfSe) and Principal Co-ordinates Analysis (PCoA) were performed online (http://huttenhower.sph.harvard.edu/galaxy/ (accessed on 10 December 2020)) and (https://www.metaboanalyst.ca/MetaboAnalyst/ModuleView.xhtml (accessed on 10 December 2020)), respectively.

### 2.7 Statistical Analysis

The indexes involved in this study were repeated three times. SPSS 25.0 statistical analysis software was used for data processing and statistical analysis. One-way analysis of variance (ANOVA) followed by Duncan analysis was used to evaluate the significant differences among all groups (*p* < 0.05). The data was expressed as mean ± SEM. The final data were analyzed by GraphPad Prism 7.

## 3. Results

### 3.1. Physiological Index

**Figure 1:**
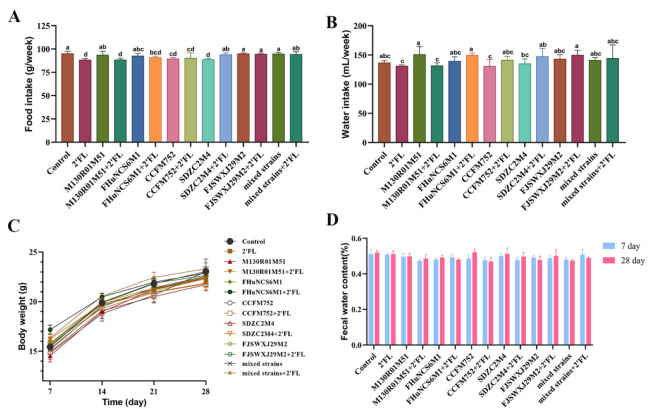
Effects of *Bifidobacterium* with different utilization of 2′FL on physiological indexes in mice (n=8). (**A**) Food intake, (**B**) water intake, (**C**) body weight, (**D**) fecal water content (%). Statistical differences were calculated by ANOVA followed by Duncan analysis and different letters indicate significant differences between the groups (*p* < 0.05).

In this study, the physiological indexes of mice were determined, including food intake, water intake, body weight, and fecal water content. Compared with the control group, 2′FL, *B. bifidum* M130R01M51 + 2′FL, *B. longum* subsp. *Infantis* CCFM752, CCFM752 + 2′FL, and *B. longum* subsp. *Infantis* SDZC2M4 treatments significantly decreased the food intake (Figure 1A). The food intake in the *B. bifidum* M130R01M51 + 2′FL group was significantly lower than that of the *B. bifidum* M130R01M51 group. Moreover, the water intake in all groups showed no significant difference compared with the control group (Figure 1B).

Compared with the control group and 2′FL group, the *Bifidobacterium* group, *Bifidobacterium* combined with 2′FL group, mixed strains, and mixed strains combined with 2′FL group had no significant effect on the body weight and fecal water content of mice (Figure 1C,D). Especially compared with mixed strains, mixed strains + 2′FL treatment showed no significant effects on food intake, water intake, body weight, and fecal water content.

### 3.2. Histological Analysis of Intestinal Morphology

**Figure 2:**
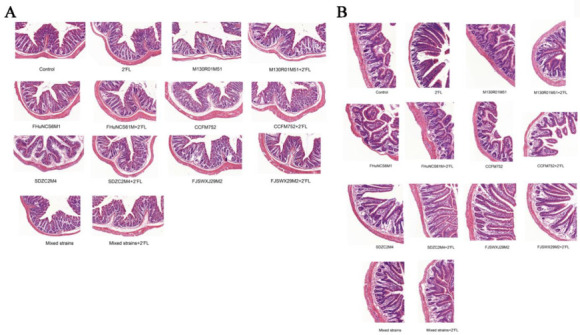
Effects of *Bifidobacterium* with different utilizations of 2′FL on morphological structure. (**A**) Colon, (**B**) ileum.

Compared with the control group, the villous epithelium of each group was intact, and the histological structure was normal. There was no significant effect on the morphology of colon tissue between the groups (Figure 2A). In terms of ileum histomorphology (Figure 2B), the villi of the small intestine in each group were arranged orderly and structurally intact without any abnormality or pathological effect. Thus, the *Bifidobacterium*, 2′FL, mixed strains, and mixed strains + 2′FL treatments had no significant effect on histological structure in the small intestine and colon.

### 3.3. Effects of Bifidobacterium on Cytokines Levels in the Colon Tissue

**Figure 3:**
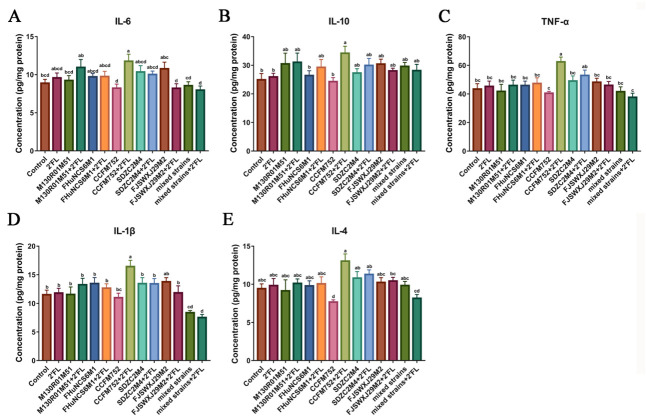
Effects of *Bifidobacterium* with different utilization of 2′FL on the concentration of cytokines in mice colon tissue (n = 8). (**A**) IL-6, (**B**) IL-10, (**C**) TNF-α (**D**) IL-1β, and (**E**) IL-4. Statistical differences were calculated by ANOVA followed by Duncan analysis and different letters indicate significant differences between the groups (*p* < 0.05).

Cytokines in the colon tissue were measured to analyze of the effects of *Bifidobacterium* and 2′FL on immunity. The results showed that compared with the 2′FL and control group, the levels of IL-6, TNF-α, and IL-1β only in the *B. longum* subsp. *longum* CCFM752 + 2′FL group were significantly increased, while the other groups showed no effects (Figure 3A,C,D). Interestingly, the *B. dentium* FJSWXJ29M2 + 2′FL treatment significantly decreased the concentration of IL-6 compared with the *B. dentium* FJSWXJ29M2 treatment.

Only the *B. longum* subsp. *Longum* CCFM752 + 2′FL treatment significantly increased the concentration of IL-10 compared with the control group, while the IL-10 in the *B. longum* subsp. *Longum* CCFM752 + 2′FL group was significantly higher than that of the CCFM752 group (Figure 3B). Only *B. longum* subsp. *Longum* CCFM752 treatment significantly decreased the content of IL-4 compared with the *B. longum* subsp. *longum* CCFM752 + 2′FL treatment (Figure 3E). Compared with mixed strains, mixed strains + 2′FL treatment showed no significant effects on IL-6, IL-10, TNF-α, IL-1β, and IL-4. Moreover, we noticed that the concentration of IL-1β was significantly decreased following the administration of a mixture of all strains, while other cytokines did not appear to differ much. This result may suggest that the mixture of all strains (+2′FL) may be responsible for the alterations that reduce inflammation in the gut.

### 3.4. Fecal Concentrations of Short-Chain Fatty Acids

**Figure 4:**
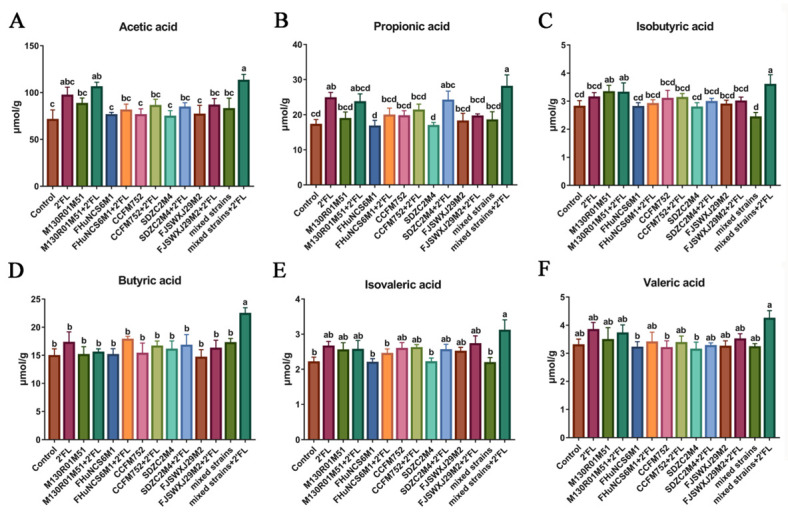
Effects of *Bifidobacterium* with different utilization of 2′FL on the concentration of short-chain fatty acid content in feces (n=8). (**A**) Acetic acid, (**B**) propionic acid, (**C**) isobutyric acid, (**D**) butyric acid, (**E**) isovaleric acid, and (**F**) valeric acid. Statistical differences were calculated by ANOVA followed by Duncan analysis and different letters indicate significant differences between the groups (*p* < 0.05).

The SCFAs content was detected in the feces of mice after *Bifidobacterium* intervention for three weeks. Compared with the control group, *B. bifidum* M130R01M51 + 2′FL and mixed strains + 2′FL treatments significantly increased the content of acetic acid, while the other groups showed no effect (Figure 4A). Compared with the single *Bifidobacterium* group, *Bifidobacterium* + 2′FL treatments did not significantly increase the content of acetic acid. Moreover, 2′FL and mixed strains + 2′FL treatments significantly increased the content of propionic acid compared with the control group, while other groups showed no effects (Figure 4B). In the single *Bifidobacterium* treatment groups, only *B. longum* subsp. *infantis* SDZC2M4 + 2′FL treatment significantly increased the propionic acid compared with *B. longum* subsp. *infantis* SDZC2M4. The isobutyric acid was significantly increased by the *B. bifidum* M130R01M51, *B. bifidum* M130R01M51 + 2′FL, and mixed strains + 2′FL treatments compared with the control group (Figure 4C). However, isobutyric acid in all *Bifidobacterium* groups showed no significant difference compared with *Bifidobacterium* + 2′FL groups. Notably, only the mixed strains + 2′FL treatment significantly increased the butyric acid and isovaleric acid compared with the control group, while the other groups showed no significance (Figure 4D,E). However, the valeric acid among all groups showed no significant difference (Figure 4F). The acetic acid, propionic acid, isobutyric acid, and butyric acid in the mixed strains + 2′FL group were significantly higher than that of the mixed strains group.

### 3.5. Modulation of Gut Microbiota by Bifidobacterium

**Figure 5:**
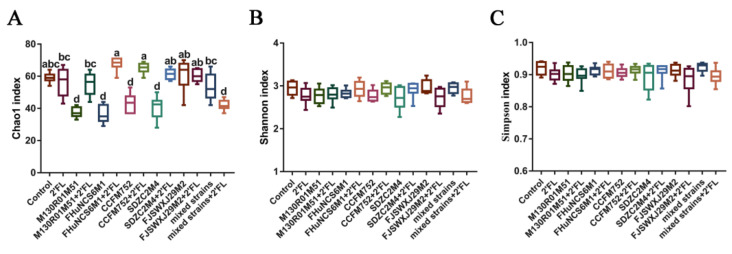
α diversity index of different groups (n=8 for each group). (**A**) Chao1 index, (**B**) Shannon index, and (**C**) Simpson index. Statistical differences were calculated by ANOVA followed by Duncan analysis and different letters indicate significant differences between the groups (*p* < 0.05).

Compared with the control and 2′FL group, the Chao1 index of the *B. bifidum* M130R01M51, *B. breve* FHuNCS6M1, *B. longum* subsp. *longum* CCFM752, *B. longum* subsp. *infantis* SDZC2M4, and mixed strains+2′FL groups decreased significantly (Figure 5A). With the benefit of 2′FL, the Chao1 index of the M130R01M51 + 2′FL, FHuNCS6M1 + 2′FL, CCFM752 + 2′FL, and SDZC2M4 + 2′FL groups was significantly higher than that of the intervention by individual bacteria. The Chao1 index in the mixed strains + 2′FL group was significantly lower than that of the mixed strains group. However, the Shannon index and Simpson index in all groups showed no significant difference (Figure 5B,C).

**Figure 6:**
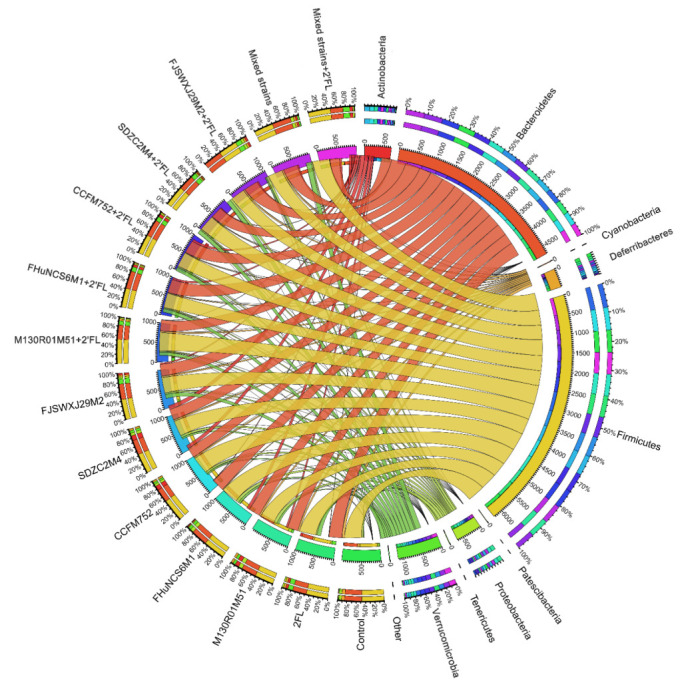
Phylum level composition of *Bifidobacterium*, *Bifidobacterium* + 2′FL, mixed strains group, and mixed strains + 2′FL groups (n = 8 for each group).

Compared with the control group, *B. bifidum* M130R01M51, *B. longum* subsp. *longum* CCFM752, and *B. longum* subsp. in*f*antis SDZC2M4 significantly increased the abundance of Actinobacteria by 7.35%, 8.44%, and 9.75%, respectively (*p* < 0.05). *B. bifidum* M130R01M51 (8.63%) significantly increased the abundance of Proteobacteria (*p* < 0.05). *B. longum* subsp. *longum* CCFM752 significantly reduced the abundance of Bacteroidetes by 22.55% compared with the control group. The relative abundance of Verrucomicrobia in the 2′FL and *B. dentium* FJSWXJ29M2 group was significantly higher than that of the control group, with an abundance of 10.87% and 11.23%, respectively (Figure 6). In addition, *B. breve* FHuNCS6M1 and *B. longum* subsp. *longum* CCFM752 combined with 2′FL significantly increased the abundance of Actinobacteria compared with the control group (*p* < 0.01). Meanwhile, the abundance of Verrucomicrobia in all groups was significantly higher than that of the control group (*p* < 0.05) (Figure 6). In addition, Bacteroides and Firmicutes were the main bacterial groups detected in mixed strains and mixed strains combined with 2′FL group (Figure 6). For mixed strains combined with the 2′FL group, the relative abundance of Verrucomicrobia was also significantly increased compared with the control group.

**Figure 7:**
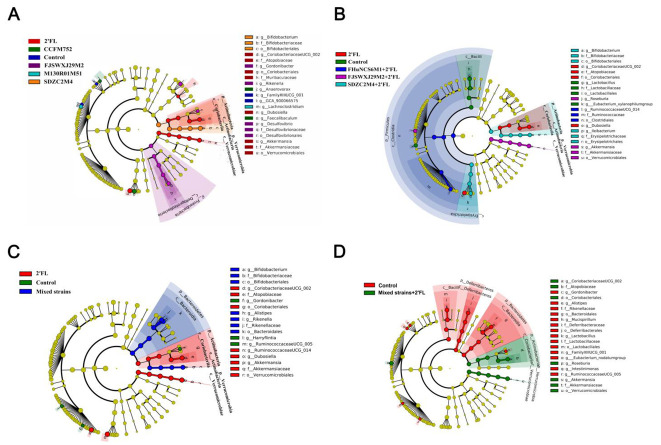
LDA analysis of *Bifidobacterium* groups (**A**), *Bifidobacterium* + 2′FL groups (**B**), mixed strains groups (**C**), and mixed strains + 2′FL groups (**D**) (n = 8 for each group).

The gut microbiota diversity among different groups and the dominant microorganisms in each group were analyzed. Dominant communities of twelve, nine, ten, and eleven taxa were found in the *Bifidobacterium*, and *Bifidobacterium* combined with 2′FL, mixed strains, and mixed strains with 2′FL group, respectively (Figure 7). Compared with the control group, the relative abundance of the selected taxa showed that the abundances of *Dubosiella*, *Coriobacteriaceae* UCG-002, and *Akkermansia* were significantly increased in 2′FL-treated mice, and the abundance of *Faecalibaculum* in the *B. longum* subsp. *longum* CCFM752 group was significantly increased.

**Figure 8:**
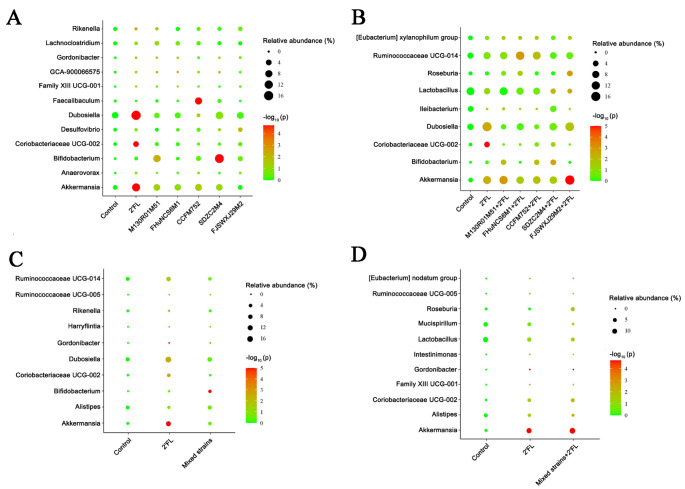
Differences in the abundance of genus levels of *Bifidobacterium* group (**A**), *Bifidobacterium* + 2′FL group (**B**), mixed strains group (**C**), and mixed strains + 2′FL group (**D**) (n = 8 for each group).

In addition, the abundance of *Bifidobacterium* in the *B. longum* subsp. *infantis* SDZC2M4 group was significantly higher than that of the control group (Figure 8A). For the *Bifidobacterium* combined with 2′FL, the *B. dentium* FJSWXJ29M2 combined with 2′FL significantly increased the abundance of *Akkermansia* compared with the control group, while other *Bifidobacterium* strains combined with 2′FL showed no significant difference (Figure 8B). In terms of relative abundance, the abundance of *Bifidobacterium* in the mixed group was significantly higher than in the control group (Figure 8C). However, the results of mixed strains combined with 2′FL group were similar to that of the 2′FL group. The abundance of *Gordonibacter* in mixed strains+2′FL group was significantly decreased, but the relative abundance of *Akkermansia* genus was significantly increased compared with the control group (Figure 8D).

**Figure 9:**
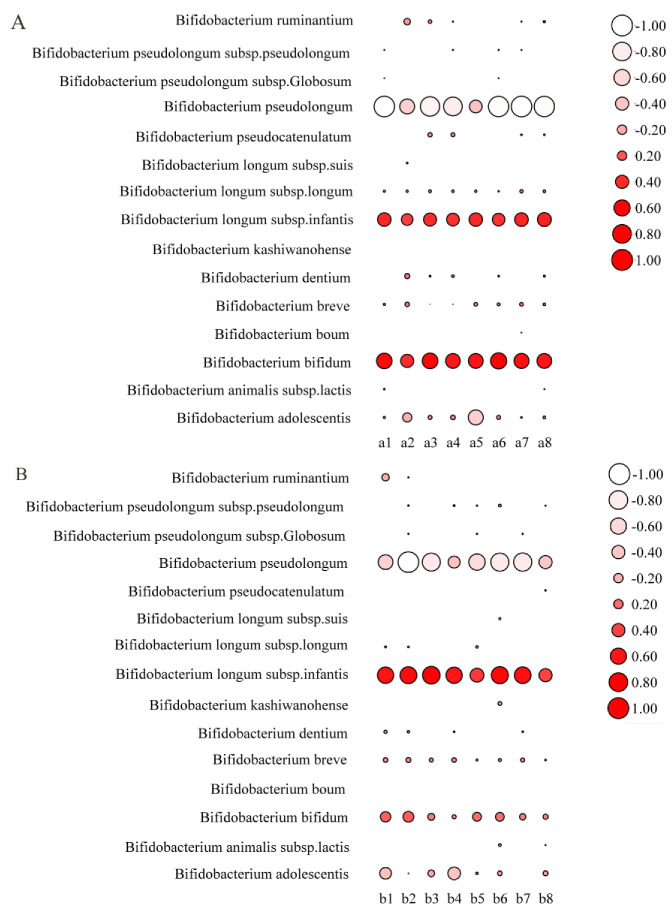
*Bifidobacterium* composition analysis of mixed strains group (**A**) and mixed strains + 2′FL group (**B**) (n = 8 for each group). a1–a8 represents different samples of the mixed strains group; b1–b8 represents different samples of the mixed strains + 2′FL group. The size of the circle represents the relative abundance. The negative value represents a decrease in the relative abundance after intragastric administration, while the positive value represents an increase in the relative abundance after intragastric administration.

The relative abundance of species of *Bifidobacterium* in the intestinal tract of mice was further analyzed. The results showed that the relative abundance of *B. longum* subsp. *Infantis* and *B. bifidum* was increased after the intragastric administration of mixed strains (Figure 9A). After the intragastric administration of mixed strains and 2′FL, the balance between *B. longum* subsp. *infantis* and *B. bifidum* appeared to be different. The relative abundance of *B. longum* subsp. *infantis* was higher than that of *B. bifidum* (Figure 9B), which may have been due to the different metabolic modes and activities of 2′FL between the two species.

**Figure 10:**
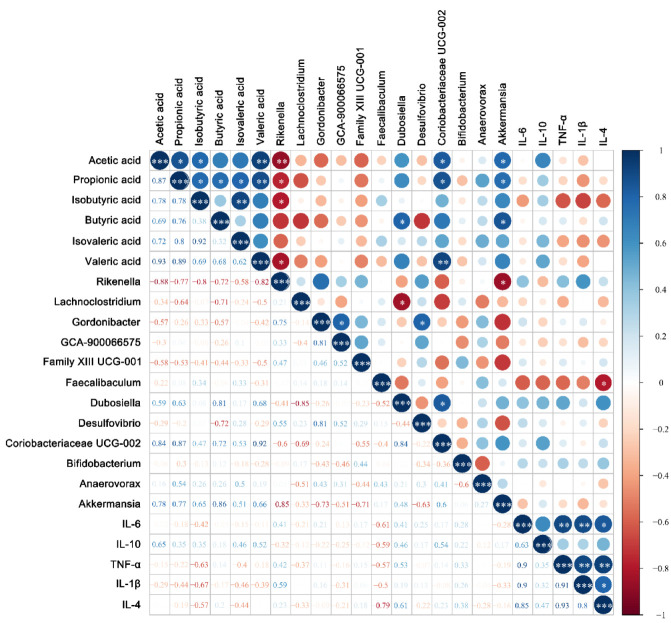
Correlation analysis of horizontal composition with short-chain fatty acids and cytokines of the fecal flora of mice in the *Bifidobacterium* group. * *p <* 0.05; ** *p* < 0.01; *** *p* < 0.001. The size of the circle represents the strength of the correlation, the negative value represents the negative correlation, and the positive value represents the positive correlation.

The correlations among differential microorganisms, SCFAs, and cytokines were also analyzed. For the *Bifidobacterium* group (Figure 10), the results showed that the relative abundance of *Dubosiella*, *Coriobacteriaceae* UCG-002, and *Akkermansia* were positively correlated with SCFAs, while the relative abundance of *Rikenella* was significantly negatively correlated with acetic acid (*p* < 0.01, r = −0.88), propionic acid (*p* < 0.05, r = −0.77), isobutyric acid (*p* < 0.05, r = −0.80), and valeric acid (*p* < 0.05, r = −0.82). For cytokines, SCFAs were negatively correlated with pro-inflammatory cytokines IL-6 and IL-1β (*p* > 0.05) and positively correlated with anti-inflammatory cytokines IL-10 (*p* > 0.05).

## 4. Discussion

2′FL can selectively promote the proliferation of *Bifidobacterium* to affect the composition of gut microbiota. Therefore, a more detailed understanding of the intestinal health effects of bifidobacteria with different utilization capacities of 2′FL alone or in combination with 2′FL is needed to truly leverage the role of probiotics in commercial formula foods and its potential to ameliorate gut microbiota imbalance in infants.

Cytokines are involved in immune responses to maintain physiological balance and prevent the occurrence of diseases. Existing studies have found that dysregulation of gut microbiota can significantly increase the levels of pro-inflammatory cytokines, such as IL-1β, TNF-α, and IL-6 [35]. However, IL-10 can inhibit the production of TNF-α and IL-6 and is an essential immune regulator in the intestinal tract [36]. At the same time, IL-4 also exacts anti-inflammatory properties in various cell models [37], regulating B and T cell growth and function, as well as IgE synthesis and release [38]. In this study, *B. longum* subsp. *longum* CCFM752 + 2′FL treatment significantly increased the levels of IL-6, IL-10, TNF-α, and IL-1β compared with the control group or *B. longum* subsp. *longum* CCFM752 treatment (*p* < 0.05), while there was no significant difference between the control group and *B. longum* subsp. *longum* CCFM752 group. It was speculated that some metabolites produced by *B. longum* subsp. *longum* CCFM752, after using 2′FL, may cause intestinal inflammation. To maintain intestinal homeostasis, the body promotes the production of anti-inflammatory factors to balance the inflammatory response. IL-1β content was significantly decreased in the mixed strains group and mixed strains combined with the 2′FL group compared with the control group. High levels of IL-1β can cause an abnormal intestinal immune response, leading to ulcerative colitis [39,40]. Therefore, the mixed *Bifidobacterium* strains have a significant stimulation of the inflammatory response of intestinal epithelial cells.

The microbial community in the gastrointestinal tract plays an important role in human physiological functions [41,42]. The difference in intestinal microbial community was measured by α diversity. Compared with the control group and 2′FL group, the Chao1 index in *B. bifidum* M130R01M51, *B. breve* FHuNCS6M1, *B. longum* subsp. *longum* CCFM752, and *B. longum* subsp. *infantis* SDZC2M4 groups was significantly decreased (*p* < 0.001). The reason may be that the intestinal environment of mice is similar to that of infants, and these bacteria may be easier to colonize in vivo than other bacteria. In addition, high doses of bacteria occupy a competitive advantage after gavage, leading to a significant decrease in the richness [12]. After 2′FL intervention, the species richness of the *B. breve* FHuNCS6M1 and *B. longum* subsp. *longum* CCFM752 combined with 2′FL groups (*p* < 0.01, *p* < 0.05) was significantly increased. The results of the Shannon index and Simpson index showed no significant difference among groups; hence, it was speculated that bifidobacteria had no obvious influence on community diversity, which was consistent with previous report [33]. However, the mixed strains combined with 2′FL showed significantly lower species abundance and evenness values than the control group, which was consistent with the previous result [43], which showed that the intervention of lactose reduced the richness and diversity of intestinal microflora in C57BL/6J mice. For the mixed strains group, the α-diversity index did not significantly change (*p* < 0.05); hence, the mixed group of multiple bifidobacteria strains can promote the balance of gut microbiota. β-diversity is a quantitative analysis of the degree of difference in intestinal species distribution, and we speculated that intestinal homeostasis is maintained by regulating gut microbiota under the influence of 2′FL [44,45].

The abundance of *Faecalibaculum* in the *B. longum* subsp. *longum* CCFM752 group was significantly increased and most of the strains could produce SCFAs. It has been shown that SCFAs are closely related to the improvement of type II diabetes. The abundance of *Bifidobacterium* was higher in *B. longum* subsp. *infantis* SDZC2M4 group. *Bifidobacterium* is the most common probiotic in Actinomycetes and plays an important role in immune regulation and intestinal health [46]. In addition, it has been reported that *B. longum* subsp. *infantis* is the dominant species in infants [27]. Therefore, we speculate that the abundance of *Bifidobacterium* in *B. longum* subsp. *infantis* SDZC2M4 group was significantly increased due to its better colonization. For *B. dentium* FJSWXJ29M2 combined with 2′FL, the abundance of *Akkermansia* increased significantly. *Akkermansia* can regulate the immune response and maintain the metabolic balance in vivo [47,48,49]. Compared with the control group, the abundance of *Bifidobacterium* in the mixed group was significantly increased, which may have been the result of a high diversity of *Bifidobacterium* fed to mice. Similarly, for the mixed strains combined with 2′FL, the relative abundance of *Gordonibacter* decreased significantly, which was consistent with previous report [50]. However, the relative abundance of *Akkermansia* increased significantly and was higher than that of the 2′FL group, but lower than that of the *B. denfidu*m FJSXWJ29M2 + 2′FL group.

Gut microbiota produce a series of complex metabolites and signaling molecules, which mediate the interaction between intestinal microorganisms and the host. Different metabolites will be produced after gut microbiota changes and may have anti-inflammatory or pro-inflammatory activities [51]. The combination of probiotics and prebiotics can regulate the production of SCFAs [52], and we found that *B. bifidum* M130R01M51 combined with 2′FL can increase the content of acetic acid and isobutyric acid in the intestinal tract. Acetic acid and propionic acid have beneficial effects on host immune regulation [53,54]. It remains unclear whether *B. bifidum* M130R01M51 and *B. longum* subsp. *infantis* SDZC2M4 can increase the content of acetic acid and propionic acid in the intestinal tract through the use of 2′FL, and this needs further experimental verification. 2′FL can promote the production of SCFAs. Thus, it is very important to supplement 2′FL in infants for intestinal health and immune system maturation [55]. Mixed bifidobacterial strains can affect the concentration of SCFAs in the intestinal tract, especially in combination with 2′FL, which was better than the single *Bifidobacterium* strain combined with 2′FL.

## 5. Conclusions

In the present study, we found that *Bifidobacterium* combined with 2′FL significantly increased the species richness of the gut microbiota, and specific strains could increase the contents of SCFAs. The results will help us to understand the benefits of bifidobacteria with the capacity to utilize 2′FL, which is very important for the future development of synbiotics with good functional characteristics, safety, and suitability for infants.

## Figures and Tables

**Table 1:** Groups and schedules of the animal experiments.

Groups	0–7 Days	8–28 Days	29 Days
Control	Period for adaptation	0.9% normal saline	Euthanasia was performed and samples were taken and various indicators were tested
2′FL		0.5 g/mL 2′FL	
*B. bifidum M130R01M51*		5 × 10^9^ CFU/mL *Bifidobacterium*	
*B. breve* FHuNCS6M1			
*B. longum* subsp. *longum* CCFM752			
*B. longum* subsp. *infantis* SDZC2M4			
*B. dentium* FJSWXJ29M2			
*B. bifidum M130R01M51* + 2′FL		5 × 10^9^ CFU/mL *Bifidobacterium* and 0.5 g/mL 2′FL	
*B. breve* FHuNCS6M1 + 2′FL			
*B. longum* subsp. *longum* CCFM752 + 2′FL			
*B. longum* subsp. *infantis* SDZC2M4 + 2′FL			
*B. dentium* FJSWXJ29M2 + 2′FL			
mixed strains *		5 × 10^9^ CFU/mL mixed *Bifidobacterium*	
mixed strains + 2′FL		5 × 10^9^ CFU/mL mixed *Bifidobacterium* and 0.5 g/mL 2′FL	

* The mixed strains consisted of five species of *Bifidobacterium* in a ratio of 1:1:1:1:1.

## Data Availability

Not applicable.
